# Registered Nurse's Competency To Screen Dysphagia Among Stroke Patients: Literature Review

**DOI:** 10.2174/1874434601812010184

**Published:** 2018-08-31

**Authors:** Hana M. Abu-Snieneh, Mohammad Y.N. Saleh

**Affiliations:** 1 School of Nursing, The University of Jordan, Amman 11942, Jordan; 2 Clinical Nursing Department, The University of Jordan, Faculty of Nursing, Amman-Jordan

**Keywords:** Nurse's competency, Dysphagia, Dysphagia Screening, Stroke, WHO, Global population

## Abstract

**Background:**

An increased number of elderly people in the world may lead to an increase in the incidence of stroke, which creates a burden on the country’s healthcare system. Dysphagia is the most common post stroke. Screening of dysphagia in stroke patients is serious to prevent complication linked to aspiration and inadequate hydration/nutrition.

**Objective:**

This literature review aimed to discuss registered nurses' competency to screen dysphagia among stroke patients.

**Methods:**

The keywords used were nurse's competency; dysphagia; dysphagia screening; and stroke. These keywords were entered into multiple electronic databases including CINAHL, Medline, Science Direct, Pro Quest, Pub Med, and Wiley Online Library. Aliterature search was conducted for the period2005 to 2016.Results:Seventeen studies were identified by a systematic search ofthe literature.Two parts created the body of this literature review. The first part covers the literature on the training nurses in screening dysphagia among stroke patients and benefits of screening. The second part covers nurse's competency in terms of knowledge and skills of screening dysphagia among stroke patients.

**Conclusion:**

Because the nurses have more contact with the patient, they are most likely to observe dysphagia. It is important that formal dysphagia screening protocols are routine nursing care that requires special training to practice. Trained nurses should assess their competency in terms of knowledge and skills via well-developed tool.

## 1 INTRODUCTION

The global population aged over 65 years is growing by 9 million a year, and by the year 2025 there will be more than 800 million people above 65 years of age in the world [1]. The World Health Organization (WHO) estimated that approximately one among six people will suffer a stroke at a time in their life [2] According to WHO, around 17.5 million people died from cardiovascular diseases in 2012, representing 31% of overall global deaths. From these deaths, an estimate of 7.4 million was due to coronary heart diseases and 6.7 million were due to stroke [3].

A stroke is a rapidly developing clinical focal disorder of cerebral function through symptoms durable for twenty-four hours or longer and leading to death, with no obvious reason other than the vascular source [3]. The most common symptom after stroke is dysphagia in an approximately half of new onset stroke [4]. Dysphagia is disruption of the bolus flow through the mouth and pharynx. As the function of swallowing is the safe delivery of a food bolus into the stomach, then the immediate complication of dysphagia is food entering the airway [5]. Dysphagia as a deficiency of eating and drinking through any or all of the different phases of swallowing [6].

Dysphagia affects a vast number of acute stroke patients. An estimated 20% to 50% of patients who have suffered a stroke have identifiable signs and symptoms of dysphagia [7]. A higher percentage (30% to 67%) was reported in stroke patients [8]. Dysphagia interrupts half of acute stroke patients and carries three folds to seven folds amplified hazards of aspiration pneumonia [9]. Up to 35% of deaths that may occur after a stroke are due to pneumonia [10]. The frequency of dysphagia in patients who were classified as severe dysphagia was 52%, and 28% with a high risk of aspiration. At the hospital discharge, only 2.1% of patients still had severe dysphagia [11].

### 1.2 Background

The presence of dysphagia among stroke patients substantially leads to psychosocial, medical, and economic problems [12-16]. Aspiration pneumonia, dehydration, malnutrition, and noteworthy weight loss are examples of medical complications of dysphagia. Dysphagia not only escalates morbidity and mortality rate after stroke, but also significantly affects the quality of stroke patients’ life when it is impossible to share their meals with family and friends and may also increase the health care costs and patients’ hospital length of stay [2, 4, 14-24].

The Joint Commission's Disease-Specific Stroke Performance Measure [25] stated “dysphagia screening should be performed on all ischemic and hemorrhagic stroke patients before being given anything by mouth, including food, fluids, or oral medications. Therefore, the patient must remain on NPO until a dysphagia screen has been completed” (p. 24). Initial screening of dysphagia is critical to prevent future health complications and should have a high priority in the health-care practices [6, 26, 27].

Nurses play a critical role in supporting the management of patients with swallowing problems [28]. Nurses are the primary health care providers for the stroke patients with dysphagia, however, there is a need to train nurses to assess and recognize dysphagia in order to enhance stroke patients’ safety [5-7, 17, 29]. The Knowledge and skills about dysphagia disorder help nurses to identify the problem on time and refer the stroke patients for diagnosis and management to the specialist health care profession [30]. If the nurses screen dysphagia within twenty-four hours of admission, it may reduce the time that patients waste without nutrition and hydration and increase good clinical outcomes for stroke patients [2]. Nursing, specifically those who are trained in undertaking dysphagia screening have an important role in reducing adverse outcomes related to dysphagia [2]. In addition, nurses are supposed to be competent to advise physicians and Speech Language Pathologist of the dysphagia symptoms [2, 20]. Many literature reported the need for training the registered nurses on how to care for the stroke patients with dysphagia [6, 10, 26, 31-35].

### 1.3 Aim

This literature review aims to discuss registered nurses' competency to screen dysphagia among stroke patients.

## 2 METHODS

The literature search untile 2016. Additional studies were identified through the searches of literature reviews, as well as from the reference lists associated with these studies. The keywords were used in multiple combinations in order to conduct an extensive search of these databases. The keywords used were dysphagia; dysphagia screening nurse's competency; and stroke. These keywords were entered into multiple electronic databases including CINAHL, Medline, Science Direct, Pro Quest, Pub Med, and Wiley Online Library. The following are the inclusion criteria for the integrative research review:

1. A research-based study.
2. Quantitative, qualitative, mixed methods and systematic reviews.
3. Written in English language.
4. Focuses on nurses' screening of dysphagia among stroke patients.

## 3 RESULTS

The articles were obtained and reviewed based on the specific inclusion criteria established, then the authors restricted the searching strategy by selecting the full text only. The analysis of the methodological characteristics of the selected studies included the purpose, samples, study design, and the measurement. At the end of the literature search, 45 full-text articles were retrieved, the titles were screened for relevance and the abstracts were read carefully, twelve not relevant articles were removed. The remaining 33 papers were read in depth and considered to be reviewed to achieve the purpose of this study. After reviewing the titles, summaries, and fully reading the articles, 17 studies were chosen. Fig. (**1**) show PRISMA diagram for Literature Review.

The majority of the studies were published in 2010 through 2016. The type of all the selected studies were quantitative research except one which was a mixed method in single-group, pre- and post-study. Most of the selected studies were non-experimental quantitative descriptive studies (n = 8). Two studies were quasi-experimental and two were a literature review, while one study was a randomized clinical trial study. In addition, two study were implementation of project and one study was clinical evaluation. One article was a single-group, pre- and post-study with mixed methods.

Two parts created the body of this literature review. The first part covers the literature on the training nurses in screening dysphagia among stroke patients and benefits of screening. The second part of the literature review covers nurses competency in terms of knowledge and skills of screening dysphagia among stroke patients. Table **1** demonstrates themes of the studies reviewed.

### 3.1 Training Nurses in Screening Dysphagia Among Stroke Patients and Benefits of Screening

Several researchers have studied the benefits of training nurses in screening dysphagia that improve health care, reduces patient’s length of stay in the hospital, lower costs of health care, reduces patient’s medical complications, decreases the staff turnover; tensions and conflict on health caregivers, and most significantly, shrink mortality rates [6, 12, 18-20, 22, 26, 31, 35, 36]. Randomized clinical trial study investiged the effects of swallowing therapy on recovery from dysphagia on sixty dysphagia patients, concluded that this stratgy prevent complications like aspiration [12].

Correspondingly, Rhoda and Pickel-Voight, (2015) reported that stroke patients commonly experience dysphagia post stroke and early diagnosis and management is an important prerequisite for recovery from stroke during the rehabilitation phase [35], Edmiaston, Connor, Steger-May, Ford, (2014) also reported that there was no pneumonia incidence during implementation of the screening dysphagia [36]. Consistently, Tanton, (2010) found that nearer identification of swallowing problems, leaded rapidly to start care pathway of dysphagia, consequently decrease risk of complication [6].

Chung, Chen, and Lee, (2013) implemented a project for nurses to enhance the accuracy and the level of understanding of the swallowing screening test and its implementation, their results reveal successfully raised the accuracy and the awareness level of swallowing screening to 100% and the occurrence of aspiration pneumonia reduced from 19% to 7% [32]. Similar Hinchey, Shephard, Furie, Smith, Wang, amd Tonn, (2005) found that pneumonia was a weighty complication of stroke and increases mortality three-fold and they concluded that formal dysphagia screening was associated with a larger adherence rate to dysphagia screen and a considerably reduced risk of pneumonia and all formal stroke patients should be screened for dysphagia [10]. Moreover, Cichero, Heaton, and Bassett, (2009) found that dysphagia screening reduces pneumonia threefold [18]. Trained nurses showed more confidence in caring patients with dysphagia and offer more attention to the nutritional requirements of stroke patients [6, 35].

Freeland, Pathak, Garrett, Anderson, and Daniels, (2016) in their study used medical mannequins to train 32 registered nurses in stroke swallowing screening, they found that this method can be used to train and evaluate nurses for acquirement and conservation of swallowing screening competency [37]. In addition, Ilott, Bennett, Gerrish, Pownall, Jones, and Garth, (2013) used a single-group, pre- and post-study with mixed methods to evaluating a novel approach (workplace-based, blended e-learning) for registered nurses and healthcare assistants, they found that this approach was an acceptable, cost effective way of delivering essential clinical knowledge and skills about dysphagia [7].

### 3.2 Nurses Competency in Terms of Knowledge and Skills of Screening Dysphagia

A competent health care provider should complete the dysphagia screening for every stroke patient with dysphagia before giving them anything by mouth, The competency of a nurse being able to perform the dysphagia screening independently leads to effectiveness for the stroke patient outcome [22]. Competence in nursing is essential to provide safe and high-quality care for stroke patients, hence registered nurses are at the forefront of recognizing and responding to deviations in a patient’s condition which may be life-threatening. To provide a high standard care for stroke patients, nurses need new knowledge and skills through an orientation program and continual education [31].

Several researchers have described registered nurses competency in term of knowledge and skills regarding screening dysphagia [30, 31, 35, 38, 39]. McHale, Phipps, Horvath, Schmelz, (1998) conducted a study to describe the practical knowledge of expert twelve nurses. Data were collected by interviews in which nurses deliberated the written narratives; nonparticipant observations and interviews; and patient chart review. By using interpretive phenomenology, they found that greatest nurse in the study did not accomplish a complete assessment of swallowing earlier in feeding patients, which was unexpected from expert nurses as they did not implement this essential assessment [39].

Moreover, Albini, Soares, Wolf, Gonçalves, (2013) conducted a study to evaluate the knowledge about the care for dysphagia patients from the nurses through answering a structured questionnaire about dysphagia. They found that nurses had a satisfactory knowledge about the definition and complications of dysphagia, but they did not know about the stages, causes and specific care related to nutrition, medication, and hygiene in cases of dysphagia, the self-assessment reported deficiency of training in conducting some procedures to patients with dysphagia. Thus, they concluded the benefits of nurse’s continual training that focuses on screening dysphagia among stroke patients [31].

Also, Diendéré *et al*, (2016) applied standardized survey in cross-section to determine nurse's knowledge and practices regarding swallowing disorders. They found that few nurses understood the importance of screening dysphagia [38]. In order to improve care among stroke patients, Diendéré, *et al*. suggested training for nurses on screening dysphagia. Similarly, Mubeen and Butt, (2014) conducted a survey to assess the knowledge of swallowing difficulties by using a self-constructed five point likert questionnaire. Their results indicated that the nurses do not have adequate knowledge of screening dysphagia and the role of the Speeach Language Pathologists [30]. Futhermore, Rhoda and Pickel-Voight, (2015) focused on the nurses, who were the first health personnel to interact with a patient with stroke, because it is important that they are knowledgeable and skilled in the screening dysphagia. however, they found that the awareness of the nurses regarding dysphagia is weak and nurses have moderate knowledge of the signs, symptoms, and complications of dysphagia, but poor knowledge about its management by application a self-administered questionnaire with closed-ended questions [35]. See Table **1** for more information.

## 4 DISCUSSION

Patients with stroke are at risk of dysphagia because they often have decreased the level of consciousness and poorer cranial nerve function followed by facial and oropharyngeal muscle weakness [14, 28]. However, clinical signs and symptoms of dysphagia are not observable all the time and the existence of an intact gag reflex does not essentially rule out the opportunity of aspiration pneumonia [15]. The possibility of mortality was higher in patients with altered swallowing than in patients with no such alteration upon clinical evaluation [40]. Chang, *et al*. (2013) reviewed that the death certificates of patients who died with stroke were 5% due to aspiration pneumonia and 1% due to choking [41].

Dysphagia screening is a marginally invasive, pass or fail tool for quick identification of patients who need a formal evaluation of swallowing administered by a trained professional [15]. Numerous clinical tools have varying sensitivity and specificity and several dysphagia screenings are available for nurses to administer among stroke patients and validate against the instrumental assessment. Although numerous dysphagia screening tools are present, no one has high sensitivity and reliability or applied quickly with minimal training [10].

One of the main responsibilities of the registered nurses is screening dysphagia among stroke patients because they have better chances to contact with patients in clinical location and they must learn the accurate guidance of safe feeding of patients with dysphagia [37]. Additionally, nurses specifically trained in the acting screening dysphagia have a significant role in dropping adverse outcomes connected to dysphagia [4, 6, 34]. However, dysphagia screening by nurses does not shift assessment by the Speech Language Pathologists; instead, it improves the care provided to patients at risk allowing for early detection and intervention [2, 6].

Knowledge of swallowing disorders in terms of signs and symptoms is fundamental for nurses working in different hospital settings. A lack of knowledge about dysphagia can result in hazardous consequences and this can be fatal for the patient. Nurses conducted the swallowing screening test inconsistently and without a standard procedure, caused chiefly by the lack of knowledge and skills on dysphagia screening, and the absence of training programs [32]. Several researchers emphasis on nurses for training the dysphagia screening [30, 31, 33, 35, 38, 39].

Training is a process by which someone has acquired the skills that are needed for a profession. Teaching enhances any knowledge and skills regarding the specific competency to improve one's capability and performance in order to increase productivity [42]. The literature supports the need for screening of dysphagia because of frequent complications of post-stroke can be detected by nurses *via* applying the appropriate valid tool [26, 31, 33, 35]. There were medical, psychological, and economic benefits for screening dysphagia among stroke patients [6, 12, 18-20, 22, 26, 31, 35, 36].

The patient needs come to be more complicated and nurses should attain essential competencies to deliver high-quality care. Care of patients with dysphagia is still a challenge because of the need for proper management of care and an interdisciplinary approach involving the Speech Language Pathologists, nurses, and medical experts [20, 25]. To add more effects in the identification of dysphagia among stroke patients in order to decrease the consequence of dysphagia, nurses ought to assess their competency using the well-developed tool in identifying the level of expertise required by their anticipated role related to their job and should be trained to that level [43].

Competency defined as the capability to do something efficiently and successfully [44]. Likewise, competency preferred outcome from an integration of knowledge, skills, abilities, judgment, and performing successfully at an expected level of nursing education and professional development [45]. Although there is no universal definition of competency [46], registered nurses must continually reassess their competencies and identify needs for additional knowledge and skills. Measuring registered nurses competencies to determine if a nurse holds the ability to perform specific tasks on clinical skills, knowledge, education, and experience in the by competency assessment tools [47]. Accordingly, healthcare organizations are anticipated to assess and continue frequently registered nurses’ clinical competencies as quantity of work to achieve the top patient care.

In the acquisition and development of a skill, a nurse passes through five levels of proficiency: novice; advanced beginner; competent; proficient; and expert [48]. In 2006, an Inter-Professional Dysphagia Competence Framework [43] was comprehensively planned for the nurses and other health care professionals to provide more effectiveness in the documentation of people with swallowing dysfunction to provide a steady approach in training and educating them. There are five levels of the Inter-Professional Dysphagia Competence Framework and their competences (Awareness; Assistant dysphagia practitioner; Foundation dysphagia practitioner; Specialist dysphagia practitioner; and Expert/consultant dysphagia practitioner) started from those who needed awareness on the presenting signs and symptoms of dysphagia and those associated with health risks to demonstrate skilled procedures with higher theoretical knowledge establishment on research/best practice. Low competence on registered nurse results spread the morbidity and mortality among stroke patients [47].

## 5 LITERATURE GAP

Dysphagia screening is a key process in the care of patients with post-stroke. All studies agreed the benefit of screening dysphagia among stroke patients. However, there was currently no agreement regarding the best approach to dysphagia screening [8, 20]. Disagreement exists regarding what tool should be used for screening and who should administer the screening [20]. All studies addressed the benefits of training nurses in screening for dysphagia among stroke patients in this literature review. In addition, various studies have created different protocols for a nurse in order to conduct a dysphagia screening, but there was no one universally utilized dysphagia screening in the hospitals [20]. There are many screening methods, but all have limitations, especially when used with acutely ill patients [6].

Publications about screening dysphagia among stroke patients started before 17 years and the methods used on the instruments were questionnaires and observation of patient’s clinical signs and symptoms [49]. Most of the studies described registered nurse competency in terms of knowledge and skills by descriptive exploratory design with purposive sampling to describe and assess knowledge about the care of the patients with dysphagia with small sample size [30, 31, 35]. But little of them investigated the practice of nurses [37-39]. Minor studies used quazi-experimental [7, 18, 37] and Randomized clinical trial study [12]. Most of the researchers concluded that nurses do not have adequate knowledge and practice in different aspects of dysphagia and screening dysphagia nurses should assess their competency in terms of knowledge and skills in well-developed tool and different evaluation tools exist. Most of the studies used self administered structured questionnaire to evaluate knowledge, while another study evaluated practical knowledge by written narratives; nonparticipant observations and interviews; and patient chart review [39].

## 6 IMPLICATION

Competence in nursing is essential to provide safe and high quality care for stroke patients, hence registered nurses are at the forefront of recognizing and responding to deviations in patient condition which may be life- threatening. The outcomes of this review highlights the role of training in a clinical placement that may be an area of research to provide sufficient regular training for a registered nurse. Educational strategies that ease the development of nurses’ knowledge, skills, and competencies in managing patient are vital. Integration of theory and practice in nurse student's curricula is important for developing nurse competence.

The current review is unique and elicit for a new body of knowledge and evidence about screening dysphagia among stroke patients in the hospitals and inform health care providers and administrators of the importance of screening dysphagia prior to initiating the oral intake of fluids or food utilizing a simple valid reliable screening tool. In addition, this review may be a platform for future setup of policies and guidelines for the health care providers to screen dysphagia among stroke patients.

## CONCLUSION

In stroke patients, dysphagia is frequent and present alarming symptom that needs urgent attention. Early screening of dysphagia is essential to prevent future health complications and should have a high priority in the health-care practices Nurses should be aware of the signs and symptoms of dysphagia to enable them recognized patients with dysphagia. Nurses, clinicians, and hospital administrators need to support teaching and training of nurses who take care of dysphagia stroke patients. Nurses need to develop their competencies on dysphagia screening based on evidence. Confirming competence in nursing practice should be a high priority for staff development departments in hospitals.

## Figures and Tables

**Fig. (1):**
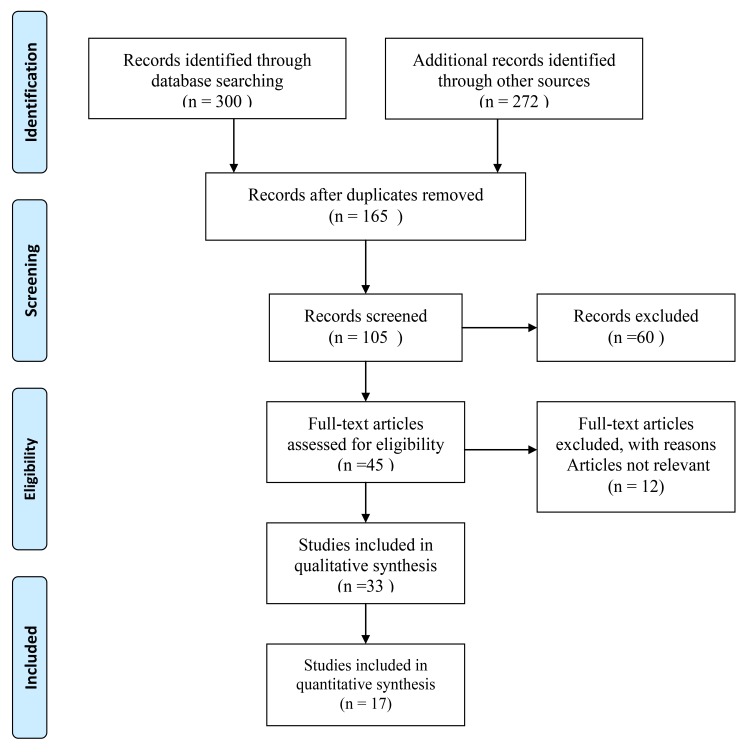
Cross section of E-TCS.

**Table 1:** The themes of the studies reviewed.

Authors/ year	Title	**Purpose**	**Design/Measurements**	**Sample**	**Results/ Conclusion**
Benefits of dysphagia screening and training nurses in screening dysphagia among stroke patients					
Tanton M. (2010)	Developing a screening tool and training package to identify dysphagia in all settings	Develop screening tool and training package to identify dysphagia	Implementation of project	Nurses	Developed an observational screening tool for nurses to ensure early and accurate identification of dysphagia and designed a training package
Ilott I, Bennet B, Gerrish K, Pownall S, Jones A, Garth A. (2013)	Evaluating a novel approach to enhancing dysphagia management: workplace-based, blended e-learning.	Evaluate the learning effect and resource use cost about dysphagia	A single-group, pre- and post-study with mixed methods.	22 Registered nurses 10 healthcare assistants	Workplace-based, blended e-learning was an acceptable, cost effective way of providing essential clinical knowledge and skills about dysphagia
Chung H L, Chen I L, Lee H L. A (2013)	A Program to Improve Accuracy Rate of Dysphagia Screening for Patients with Stroke.	Enhance the accuracy and the level of understanding of the swallowing screening test and its implementation	Implementation of project	Nurses	Implementation of the countermeasures successfully increased the accuracy and the awareness level of swallowing screening and reduced the incidence of aspiration pneumonia
Edmiaston J,Connor LT, Loehr L, Nassief A. (2010)	Validation of a dysphagia screening tool in acute stroke patients.	Design and validate a swallowing screening tool to be used by nurses.	Prospective study	Nursing staff administered tool over 300 stroke patients	The Acute Stroke Dysphagia Screen tool has important to detect dysphagia and aspiration risk.
Singh S, Hamdy S. (2006)	Dysphagia in stroke patients	Discuss identification, clinical course, pathophysiology, and treatment of dysphagia.	A Systematic Literature Review		Dysphagia carries a sevenfold increased risk of aspiration pneumonia and is an independent predictor of mortality
Hinchey J A, Shephard T, Furie K, Smith D, Wang D. Tonn S. (2005)	Formal dysphagia screening protocols prevent pneumonia	Identify constitutes an adequate dysphagia screen.	Prospectively study	15 acute care institutions	A formal dysphagia screen is associated with a higher adherence rate to dysphagia screens and a significantly decreased risk of pneumonia. .
Edmiaston J, Connor L T, Steger-May K, Ford A L. (2014)	A simple bedside stroke dysphagia screen, validated against video-fluoroscopy, detects dysphagia and aspiration with high sensitivity	Assess the accuracy of the BJH-SDS.	Prospective study	225 acute stroke patients	High sensitivity and specificity of the screen to detect dysphagia No increase in pneumonia was identified during implementation of the screen (p=0.33).
Freeland,T.R., Pathak, S., Garrett, R.R., Anderson, J., Daniels, S. K. (2016)	Using Medical Mannequins to Train Nurses in Stroke Swallowing Screening.,	Determine feasibility using of medical simulation mannequins as a training component	Quasi experimental	32 registered nurses	Simulation training using medical mannequins can be used to train and evaluate nurses for attained and keep competency of swallowing screening
Bakhtiyari J, Sarraf P, Nakhostin-Ansari N, *et al* . (2015)	Effects of early intervention of swallowing therapy on recovery from dysphagia following stroke.	Investigation on the effects of swallowing therapy on recovery from dysphagia following stroke.	Randomized clinical trial study.	Sixty dysphagia patients	Effective onset time of swallowing therapy after stroke on recovery from dysphagia and prevention of complications like aspiration pneumonia
Cichero J A Y, Heaton S, Bassett L. (2009)	Triaging dysphagia: nurse screening for dysphagia in an acute hospital	Develop a dysphagia screening tool to triage patients and evaluate tool reliability, evaluate nursing compliance then develop a robust dysphagia training programme	Prospective, quasi experimental	Nurses	The dysphagia screening is a quick and excellent tool for triaging individuals with dysphagia. Training is critical to successful screening.
Matesic E. (2010)	Evaluation of Testing and Implementation of Evidence-based RN Bedside Swallow Screen for Dysphagia: A Clinical Practice Change”	Establishing a bedside swallow screen	Evaluation study	Nurses	Acute stroke patient can safely receive medications and nutrition by mouth because of swallow screen Organizations must funding such evidence-based practice consequential in cost-benefit and improve care quality
Etges C L, Scheeren B, Gomes E, Barbosa L R. (2014)	Screening tools for dysphagia: a systematic review.	To perform a systematic review of screening instruments for dysphagia available in the literature.	Systematic review		Screening instruments in dysphagia are varied and have been developed for diverse audiences with the different aim.
Nurse's competency in terms of knowledge and skills of screening dysphagia among stroke patients.					
Mubeen R, Butt A K. (2014)	Knowledge of Dysphagia, It’s Screening among Nurses and Awareness of Role of Speech and Language Pathologist in Dysphagia	Establish the knowledge of dysphagia and its screening among nurses	A non-experimental, descriptive survey	80 Purposive convenient nurses	Lack of knowledge of nurses regarding dysphagia
Albini R M N, Soares V M N, Wolf A E, Gonçalves C G O. (2013)	Knowledge of Nursing Professionals about the Care to Dysphagic Patients in Intensive Care Units,	Evaluate the knowledge about the care dysphagic patients and knowledge about dysphagia, and its implications on intensive care unit from the nurses	A quantitative, descriptive and comparative study	Nurses	Adequate knowledge about the definition and complications of dysphagia, lack knowledge about the stages, causes and care related to nutrition, medication and hygiene. lack of training in conducting procedures to dysphagic patients
Rhoda A, Pickel-Voight A. (2015)	Knowledge of nurses regarding dysphagia in patients post stroke in Namibia’	Determine the knowledge and factors associated with knowledge of nurses regarding dysphagia in stroke patients.	A quantitative descriptive survey	188 convenient Nurses	Nurses have a moderate knowledge of the signs, symptoms, and complications of dysphagia, but poor knowledge about its management.
Diendéré J. *et al* . (2016*)*	Knowledge and practice concerning swallowing disorders in hemiplegic patients on nurses of Bobo–Dioulasso urban primary health care centers in Burkina Faso	Assess knowledge and practices regarding swallowing disorders	Cross-sectional study	125 Nurses	Few nurses had connected swallowing disorders and potential complications. Practices varied, but most were not in accord with what are recognized as good strategies for swallowing disorders screening and management.
McHale J, Phipps M, Horvath K, Schmelz J. (1998)	Expert nursing knowledge in care of patients at risk of impaired swallowing.	Describe the practical knowledge of expert nurses when they assess and feed patients at risk of impaired swallowing.	Descriptive exploratory study	12 Purposive nurses	Most nurses in the study did not perform a complete assessment of swallowing before feeding their patients.
